# Non-invasive Ventilation for Children With Chronic Lung Disease

**DOI:** 10.3389/fped.2020.561639

**Published:** 2020-11-11

**Authors:** Emine Atag, Uros Krivec, Refika Ersu

**Affiliations:** ^1^Division of Pediatric Pulmonology, Medipol University, Istanbul, Turkey; ^2^Division of Pediatric Pulmonology, University Children's Hospital, University Medical Centre, Ljubljana, Slovenia; ^3^Division of Pediatric Respirology, Children's Hospital of Ontario, University of Ottawa, Ottawa, ON, Canada

**Keywords:** children, chronic lung disease in childhood, Non-invasive Ventilation (NIV), cystic fibrosis, Bronchopulmonary Dysplasia (BPD)

## Abstract

Advances in medical care and supportive care options have contributed to the survival of children with complex disorders, including children with chronic lung disease. By delivering a positive pressure or a volume during the patient's inspiration, NIV is able to reverse nocturnal alveolar hypoventilation in patients who experience hypoventilation during sleep, such as patients with chronic lung disease. Bronchopulmonary dysplasia (BPD) is a common complication of prematurity, and despite significant advances in neonatal care over recent decades its incidence has not diminished. Most affected infants have mild disease and require a short period of oxygen supplementation or respiratory support. However, severely affected infants can become dependent on positive pressure support for a prolonged period. In case of established severe BPD, respiratory support with non-invasive or invasive positive pressure ventilation is required. Patients with cystic fibrosis (CF) and advanced lung disease develop hypoxaemia and hypercapnia during sleep and hypoventilation during sleep usually predates daytime hypercapnia. Hypoxaemia and hypercapnia indicates poor prognosis and prompts referral for lung transplantation. The prevention of respiratory failure during sleep in CF may prolong survival. Long-term oxygen therapy has not been shown to improve survival in people with CF. A Cochrane review on the use NIV in CF concluded that NIV in combination with oxygen therapy improves gas exchange during sleep to a greater extent than oxygen therapy alone in people with moderate to severe CF lung disease. Uncontrolled, non-randomized studies suggest survival benefit with NIV in addition to being an effective bridge to transplantation. Complications of NIV relate mainly to prolonged use of a face or nasal mask which can lead to skin trauma, and neurodevelopmental delay by acting as a physical barrier to social interaction. Another associated risk is pulmonary aspiration caused by vomiting whilst wearing a face mask. Adherence to NIV is one of the major barriers to treatment in children. This article will review the current evidence for indications, adverse effects and long term follow up including adherence to NIV in children with chronic lung disease.

## Introduction

Non-invasive ventilation (NIV) is the application of ventilatory support via a non-invasive interface instead of an endotracheal tube or tracheostomy. Following early mechanical ventilation applications as negative pressure ventilation in the 1930s, the use of NIV in children has increased exponentially around the world in recent years (1–3). There are many studies showing the benefits and superiority of NIV in comparison with invasive ventilation via tracheostomy in terms of quality of life, health care costs, and morbidity (4, 5).

There are two main modes to provide positive airway pressure support. Continuous positive airway pressure (CPAP) provides constant positive pressure throughout the entire respiratory cycle, which prevents airway closure. Therefore, CPAP does not assist spontaneous inspiration of the patient, CPAP improves gas exchange and oxygenation by increasing functional residual capacity (FRC). Bilevel positive airway pressure (BIPAP) delivers a pre-set positive pressure at inspiration as well as a background positive expiratory pressure. BIPAP improves ventilation and gas exchange more effectively than CPAP alone by increasing tidal volume as well as FRC (6).

Normal spontaneous ventilation requires a balance between neurologic mechanism controlling ventilation (respiratory drive) together with respiratory muscle power and respiratory load. Conditions that disrupt this respiratory balance are the main indications for NIV such as upper airway obstructions, lower airway obstructions due to chronic lung disease, weakness of respiratory muscles and disorders of central drive (4).

Advances in medical care and expansion of supportive care options have contributed to the survival of children with complex disorders including children with chronic lung disease (CLD). Asthma, bronchopulmonary dysplasia (BPD), cystic fibrosis (CF), non-CF bronchiectasis, bronchiolitis obliterans (BO), interstitial lung disease (ILD) and CLD in children with HIV cause airway and parenchymal inflammation, decreased lung compliance leading to chronic airflow obstruction, tachypnea and increased work of breathing. When the respiratory load increases, the work of the respiratory muscles also increases to avoid respiratory failure. This causes shortness of breath, exercise intolerance, growth failure and if ventilatory support is inadequate, it can be associated with hypoxemia and hypercapnia leading to pulmonary hypertension, cor pulmonale, and right heart failure. NIV is not needed for all CLD as in the case of asthma vast majority of patients do not need NIV.

In obstructive lung diseases such as CF, airflow obstruction, and parenchymal lung damage cause increased pulmonary resistance and decreased compliance due to parenchymal fibrotic change resulting in increased respiratory load. Respiratory muscle weakness caused by malnutrition and chronic pulmonary hyperinflation may also contribute to impaired respiratory function (7).

The respiratory load is increased in ILD due to several mechanisms. Children with ILD typically have a rapid shallow breathing pattern as a result of an increase in central drive, due to vagally mediated response to decreased lung compliance. Lung compliance is decreased in children with ILD as a result of extracellular matrix deposition. In addition, increased lung resistance and work of breathing result in increased respiratory rate. An impairment in gas exchange due to ventilation-perfusion mismatch and reduced diffusion capacity also contribute to the increase in respiratory rate and respiratory load (8, 9).

NIV is not a universal requirement for patients with chronic lung disease. The primary aims of NIV in children with CLD are to improve minute ventilation, increase alveolar recruitment and improve compliance which in turn unload respiratory muscles and reduce work of breathing (4). Non-invasive ventilation is also an effective treatment for nocturnal hypoventilation. By delivering a positive pressure during patient's inspiration, NIV is able to reverse nocturnal alveolar hypoventilation in patients who experience hypoventilation during sleep, such as patients with chronic lung disease (2, 3). Several physiologic mechanisms are responsible for hypoventilation during sleep. Compared to wakefulness, there is a decrease in central drive and chemoreceptor sensitivity affecting ventilatory response to hypoxia and hypercapnia during sleep. Sleep has also adverse effects on respiratory muscle function. While the function of the diaphragm is preserved, there is a decrease in the tone of the accessory respiratory muscles. Additionally, decreased FRC, increased ventilation-perfusion mismatch, increased airflow resistance also play a role in the development of nocturnal hypoventilation (10). Children with moderate-severe CF lung disease have lower oxygen saturations and more frequent desaturation episodes during sleep compared to healthy children, due to adverse effects of sleep on the existing ventilation-perfusion mismatch caused by parenchymal lung disease. This decline in oxygen saturation levels is correlated with awake resting oxygen saturation, and disease severity such as FEV_1_ % predicted. Poor sleep quality, reduced sleep efficiency, and total sleep duration among children with CF also contribute to nocturnal hypoxemia in these children (11).

Furthermore, in BPD, central apneas and abnormal ventilatory responses to hypoxic and hypercarbic states due to the immaturity of respiratory drive and sleep fragmentation are common. In addition to the immaturity of respiratory drive, upper airway obstruction is also increased during sleep as a result of a smaller caliber upper airway and increased compliance of the chest wall (12, 13).

As mentioned above, there are also various changes in respiratory physiology in ILD. During sleep, decreased respiratory drive, chemosensitivity and decreased activity of respiratory muscles including the diaphragm cannot maintain alveolar ventilation. Decreased lung volumes and altered ventilation-perfusion match further increase alveolar hypoventilation in ILD (14, 15).

The use of NIV in CLD may rarely be contraindicated, ineffective, or in some cases harmful. The presence of pneumothorax in children with advanced disease is an absolute contraindication for NIV. Judicial evaluation of patients with severe cystic/cavitatic lung disease and their suitability for NIV is required. Besides, in cases with treatment failure, nasal polyps should be considered in children with CF and asthma (10). In patients with ILD, who have a low percentage of the recruitable lung, high PEEP levels may be ineffective or even harmful as a result of hyperinflation of already open lung regions (16, 17).

This article will review the current evidence for indications, adverse effects and long term follow up including adherence to NIV in children with chronic lung disease.

## Asthma

Asthma is a common chronic lung disease, characterized by chronic airway inflammation, persistent remodeling of the small airways and bronchial hyperresponsiveness. The main clinical findings are wheezing, shortness of breath, chest tightness and cough with variable expiratory airflow limitation. Regular controller therapy is required to achieve symptom control and avoid respiratory morbidity such as exacerbations (18, 19).

Asthma exacerbation occurs due to reversible, diffuse lower-airway obstruction, caused by airway inflammation and edema, smooth muscle contraction, and mucus hypersecretion. This obstruction of the lumen limits airflow and causes premature closure of the airways (20). An active expiration by continuous activation of inspiratory muscles is required to empty the lungs. Besides, increased airway resistance and air trapping make it difficult to further stretch the hyperinflated lungs for adequate inspiration. Progressive air trapping increases positive end-expiratory pressure (PEEP), also known as auto-PEEP or intrinsic PEEP. This increase is correlated with the degree of the obstruction, and results in muscle fatigue and respiratory failure, if not properly and promptly treated (21). The main initial therapies include bronchodilators, corticosteroids and oxygen supplementation. Despite improvements in the management of asthma exacerbations, some patients experience severe exacerbations not responding optimally to medical therapies (18).

NIV may be able to counteract the intrinsic PEEP by delivering an extrinsic EPAP. It maintains airway patency, which results in improvement of airflow, re-expansion of atelectatic lung segments, and a decrease in respiratory muscle load. These changes also improve ventilation–perfusion mismatch. Also, NIV can increase tidal volume by improving respiratory muscle functions (22–24).

Delivering nebulized bronchodilators via NIV also improves lung aerosol dispersion, and may increase response to bronchodilators, which may improve clinical outcomes (25, 26).

Based on this physiologic rationale, there are several studies on the NIV use for children with acute asthma exacerbations. A recent Cochrane review included the following two studies on the use of NIV in children with acute asthma exacerbation (19). Thill et al. studied the effect of NIV among 20 children with acute asthma exacerbation who were admitted to the pediatric intensive care unit (ICU) in a prospective cross-over study. Patients were randomized to receive either 2 h of NIV followed by crossover to 2 h of conventional therapy or 2 h conventional therapy followed by 2 h of NIV. NIV was associated with a decrease in respiratory rate for all patients. There was also a decrease in clinical asthma scores both in total and in each of the sub-scores. Additionally, the discontinuation of NIV was associated with an increase in respiratory rate and clinical asthma score. No adverse events related to NIV were observed during the study. Authors demonstrated that NIV improved clinic findings of respiratory distress and it may be a treatment option for the children with severe acute lower airway obstruction refractory to medical therapy (23).

The effect of early initiation of NIV in asthmatic children admitted to the ICU was investigated in a randomized controlled study by Basnet et al. NIV use for 24 h as initial treatment was compared to standard medical treatment alone. This study revealed that the improvement in clinical asthma score and respiratory functions determined by a decrease in respiratory rate and oxygen requirements were greater in the NIV group. There was also a trend to a decrease in the need for other adjunct therapies. Non-invasive ventilation was well-tolerated except for one patient (27).

Despite increased use of NIV, the evidence on the role of NIV among asthmatic patients is weak (18, 19). A study investigated outcomes of asthmatic patients admitted to the ICU revealed that the NIV use increased from 11 to 39% over 12 years. However, intubation rates remained similar despite increasing rates of NIV use (28).

The use of NIV in asthmatic patients may be considered as an add-on therapy to standard care. If NIV is tried, close monitoring of the patient is essential. Global Initiative for Asthma (GINA) recommends that NIV should not be attempted in agitated patients with asthma, and patients should not be sedated in order to receive NIV (Evidence D) (18).

## Bronchopulmonary Dysplasia

Bronchopulmonary dysplasia is the most common cause of chronic lung disease in infancy and the most common morbidity of prematurity (29). Since the first definition of the BPD, the definition is evolved over time (30). According to a workshop report, BPD is defined as oxygen need for 28 days and an assessment of respiratory support at 36 weeks' postmenstrual age was proposed for defining the severity (Table 1) (31). There is a further definition to determine the severity of the disease. Infants in room air at 36 weeks PMA are “mild,” those requiring <30% oxygen are “moderate,” and those requiring ≥30% oxygen or positive pressure ventilation are “severe” (32, 33). Bronchopulmonary dysplasia is also divided into early, evolving and established BPD. The time from birth to the 1st week of life indicates early BPD. The period between 1st week of life and 1st month of life called as evolving BPD and established BPD is considered to occur after 1st month of life (34). The current definitions of BPD still have some limitations such as the optimal timing to assessment (35). A study evaluating optimal definition of BPD to predict long-term respiratory outcomes at 18–21 months corrected age showed receiving oxygen and/or respiratory positive-pressure support at 40 weeks' PMA was most strongly associated with serious respiratory outcome among those at each of 34–44 weeks' PMA (36).

**Table 1:** Definition of BPD: Diagnostic criteria.

**Gestataional age**	**<32 week**	**≥32 week**
Time point of assessment	36 week PMA or discharge to home, whichever comes first	>28 day but <56 day postnatal age or discharge to home, whichever comes first
**Treatment with oxygen** **>21% for at least 28 d plus**		
Mild	Breathing room air at 36 week PMA or discharge, whichever comes first	Breathing room air by 56 day postnatal age or discharge, whichever comes first
Moderate	Need for <30% at 36 week PMA or discharge, whichever comes first	Need for <30% at 56 day postnatal age or discharge, whichever comes first
Severe BPD	Need for ≥30% oxygen and/or positive pressure (PPV or NCPAP) at 36 week PMA or discharge, whichever comes first	Need for ≥30% oxygen and/or positive pressure (PPV or NCPAP) at 56 day postnatal age or discharge, whichever comes first

*BPD, bronchopulmonary dysplasia; NCPAP, nasal continuous positive airway pressure; NIH, National Institutes of Health; PMA, postmenstrual age; PPV, positive pressure ventilation. Source: Jobe AH, Bancalari E. Bronchopulmonary dysplasia. Am J Respir Crit Care Med 2001;163(07):1723–1729*.

Despite significant advances in neonatal care over the recent decades, the incidence of BPD has not diminished. The reported rate varies between 30 and 59% depending on the gestational age; babies who are <1,000 g, and <28 weeks of gestation, accounting for most of the cases (32). Most affected infants have mild disease and require a short period of oxygen supplementation or respiratory support. However, severely affected infants can become dependent on positive pressure support for a prolonged period. The severity of BPD is also inversely correlated with gestational age. The incidence of severe BPD is reported as 16 and 25% in infants born <32 and <27 weeks, respectively, with a male predominance. Although the main predisposing factor is prematurity, many other factors such as antenatal inflammation, intrauterine growth retardation, maternal smoking, male gender and genetic factors are associated with the increased risk of BPD and poor respiratory outcomes (37).

Pulmonary inflammation, oxidative stress, mechanical trauma to the immature lungs cause disrupted alveolar and vascular growth leading to fewer and larger alveoli, decreased pulmonary vasculature, and variable smooth muscle proliferation (38, 39). These changes generate respiratory morbidity and long-term impairment in lung functions in BPD survivors. Preterm infants with BPD experience more wheeze episodes, require inhaled medications more frequently and hospitalizations during the 1st years of life (40, 41). As infants with BPD grow, there is a decrease in total pulmonary resistance, an increase in pulmonary compliance, and an improvement in the ventilation-perfusion ratio, resulting in decreased respiratory rate, increased tidal volume, and improved oxygenation (42). However, cross-sectional and longitudinal studies evaluating respiratory functions following the development of the BPD revealed a significant airway obstruction from the infancy tracking into adulthood (43–46). These results support that BPD survivors may not achieve their optimal airway growth.

There is no single management strategy for the wide spectrum of clinical presentations of BPD and care must be individualized. Gentle respiratory support, titration of supplemental oxygen are mainstay of preventive respiratory management. In case of established severe BPD, respiratory support with non-invasive or invasive positive pressure ventilation is required (34, 37).

Invasive mechanic ventilation is a well-known risk factor in the development of BPD. To decrease the volu- and barotrauma related lung injury NIV strategies are introduced in neonatal respiratory management, and it has become increasingly popular over time. NIV allows infants with BPD to wean from invasive mechanical ventilation and prevents the recurrence of respiratory failure requiring re-intubation (47).

Non-invasive respiratory support has been used after extubation to reduce extubation failures, or as a primary modality for premature infants with respiratory distress syndrome, and also to prevent late respiratory morbidity/BPD for premature babies (47).

Continuous PAP (CPAP) is the first and most commonly used mode of positive airway pressure (PAP) therapy for neonatal respiratory support (48). Continuous PAP provides alveolar inflation and prevents atelectasis. The physiological benefits of CPAP such as stabilization of chest wall and upper airway, reduction of lung resistance and improvement of tidal volume, oxygenation, and functional residual capacity increase the success of extubation of preterm infants (9). The use of CPAP significantly reduced the need for invasive mechanical ventilation but the rates of CPAP failure are high. On the other hand, studies evaluating effects of CPAP on preventing BPD, showed that use of nasal CPAP did not significantly reduce the rate of BPD and death compared with the infants who were intubated early and received surfactant (49, 50).

Nasal intermittent positive pressure ventilation (NIPPV) is an alternative strategy to CPAP. It is a type of NIV delivered via nasal prongs using a mechanical ventilator. NIPPV provides mandatory ventilation at a preset rate using short inflation times, and two different airway pressure levels similar to those used with invasive ventilation. Nasal intermittent positive pressure ventilation can be applied in a synchronized mode (SNIPPV) or can be delivered independent of the infant's breathing efforts. The synchronization of NIPV is challenging (33, 51). A meta-analysis examining the benefits of early NIPPV vs. CPAP showed significantly reduced risk of extubation failure among infants treated with NIPPV. However, a meta-analysis found no difference in the risk of BPD between NIPPV and CPAP (52).

The comparison of SNIPPV vs. NIPPV, according to a meta-analysis, showed that SNIPPV was superior to NIPPV in terms of the need for re-intubation up to 1 week after extubation. However, there was no difference in the rates of the development of BPD between these two interventions (53). Furthermore, a recent systematic review comparing ventilation strategies including NIPPV among preterm infants also showed no differences in outcomes of BPD between NIPPV and other ventilatory strategies (54).

Bilevel positive airway pressure is another form of NIV and has a similar mechanism to NIPPV. BIPAP also provides two levels of positive pressure (PIP and PEEP) at preset intervals. The pressure delivered by BIPAP is lower than NIPPV. Additionally, BIPAP provides longer inspiratory times and lower cycle rates than NIPPV and allows spontaneous breathing in contrast to NIPPV (55). Possible benefits include tiny actual breaths delivered from the small change in pressure and reflex “triggering” of spontaneous breaths via stimulation. Also, since high pressures can be used, it is better at preventing reintubation compared to CPAP (50). However, there are few studies investigating the use of BIPAP among preterm babies, and the results are inconclusive. In the first of the randomized studies in the literature, BIPAP was found to be superior to CPAP in respiratory support time, while no difference was found in another study (56, 57). In a later retrospective study, the duration of intubation was shorter in the group of <32 weeks old infants using BiPAP. The frequency of BPD was similar in all of these studies (58). Studies comparing BiPAP to NIPV also revealed no difference between the rates of the development of BPD between the two NIV modes (59).

As mentioned above, despite the common use of NIV modes, none of the NIV modes reduced the frequency of BPD. A study investigating practical differences in the use of NIV strategies across Canadian neonatal intensive care units showed that CPAP was used in all centers, but the rate of BiPAP use was 79%. According to this study, NIV practices were performed only in 61% of all cases based on local guidelines. There was also a significantly high variability in terms of settings and interfaces used. The authors of this study concluded that the long-term results of NIV would be better with optimized standardized treatments rather than various clinical practices and underlined the need for universal evidence based-guidelines on the use of NIV (60).

The data of patients with BPD are quite limited in the studies evaluating patients using long-term NIV in the literature and invasive ventilation is still a common approach for patients with BPD requiring long-term respiratory support.

## Cystic Fibrosis

Pulmonary involvement is the primary cause of morbidity and mortality in patients with CF. Mucus plugging, chronic inflammation, and bronchiectasis cause airflow obstruction and parenchymal damage, followed by a progressive deterioration in lung functions. Despite the improvement in CF care, chronic respiratory failure, and end-stage lung disease develop in most of the patients (61).

According to physiological studies there is an increase in respiratory load in CF patients. Respiratory muscle load, which is assessed by the esophageal pressure time products and diaphragmatic pressure time products and the elastic and the total work of breathing is increased in CF patients. This increase in respiratory load is also correlated with the progression of pulmonary function decline. However, respiratory muscle activity is preserved and central drive is normal or increased in patients with CF. Despite the preserved respiratory muscle activity and normal or increased central drive, alveolar hypoventilation occurs in CF patients with the worsening of lung disease, especially during conditions requiring an increase in minute ventilation (exercise, respiratory infections) due to the inability of the patients to increase their respiratory rates any further (62).

Central drive and chemoreceptor sensitivity are preserved during wakefulness in patients with mild-moderate CF lung disease, but alveolar hypoventilation characterized by an increase in partial arterial carbon dioxide pressure (PaCO_2_) and decrease in partial arterial oxygen pressure (PaO_2_) may occur during sleep. During sleep, there are physiological changes in central drive, respiratory muscle function and lung mechanics. Decrease in central drive and chemoreceptor sensitivity during sleep cause alveolar hypoventilation and a rise in PaCO_2_. Respiratory muscle function is also adversely affected, especially the accessory muscles of respiration and upper airway muscles, whereas diaphragmatic contraction is relatively preserved during sleep. Altered respiratory mechanics in terms of an increase in ventilation–perfusion mismatch, an increase in airflow resistance, and a decrease in functional residual capacity, contribute to nocturnal hypoventilation (10, 62).

With the progression of pulmonary involvement and increased respiratory load, patients develop a rapid shallow breathing pattern and subsequent daytime hypercapnia (10, 62). In addition, chronic alveolar hypoxia, hypercapnia, and endothelial dysfunction due to chronic inflammation may cause pulmonary hypertension (63, 64). Pulmonary hypertension is inversely correlated with respiratory function and nocturnal oxygen saturation. Hypoxemia, hypercapnia and pulmonary hypertension indicate poor prognosis and prompts referral for lung transplantation (65).

NIV, which acts as an external respiratory muscle, reduces the number of required respiratory efforts by the patient and reduces the muscle load for a given tidal volume during an assisted breath. These effects can reverse fatigue and reduce neural drive to prevent fatigue (66). The improvement in lung and chest compliance causes a decrease in respiratory muscle load and an increase in tidal volume, in alveolar ventilation and gas exchange (10, 67).

Implementation of NIV even for a short period (during sleep, exercise) allows improvements in alveolar ventilation and gas exchange, which is a result of the increased chest wall and lung compliance and decreased cerebrospinal fluid bicarbonate concentration increase respiratory drive (62).

There are no validated criteria for timing and patient selection for the initiation of NIV, particularly in the pediatric population, despite the physiological benefits of NIV in CF patients. According to the Cystic Fibrosis Foundation recommendations, the presence of the symptoms related to hypercarbia, and a PCO_2_ level ≥55 mmHg or; PaCO_2_ 50–54 mmHg and nocturnal desaturation, or, PaCO_2_ 50–54 mmHg and ≥2 hospitalizations in the preceding year for hypercarbic respiratory failure are the main indications for initiation of NIV (68). The European CF Society recommends NIV according to the patient's wishes for relief of dyspnea (69).

There are several studies on the use of NIV for cystic fibrosis which reported clinical indications. According to a national survey performed in France, severe respiratory exacerbation and diurnal hypercapnia were the main indications for NIV use. NIV may also be useful for patients with an accelerated fall in lung functions and its use may be proposed to prevent or treat an acute severe respiratory exacerbations. NIV use is also an option in palliative care for end-stage CF lung disease (70, 71).

There are no studies on the effectiveness of NIV in children with CF acute exacerbations, but adult studies showed good effect of NIV during exacerbations. A study investigating outcomes of adult CF patients admitted to the ICU revealed that the patients managed with NIV had lower mortality rates compared to patients receiving invasive mechanical ventilation (72). A later retrospective multicenter study investigated the outcomes of 60 ICU admissions of 42 adult CF patients. Pulmonary exacerbation was the main reason for ICU admission (67%). NIV was used in almost 75% of admissions for pulmonary exacerbations and was efficient in two-thirds of the cases. NIV success was more frequent among patients with prior home NIV (61 vs. 36%) (73).

NIV can also be used as a first-line treatment outside the ICU to prevent the worsening of acute exacerbation. In a retrospective study, cases were treated either in the pulmonary department or in the ICU depending on the severity of the acute exacerbation. The rate of NIV use was 33% (15 out 45) and 61% (14 out 23) in the pulmonary department and ICU, respectively. There were no deaths reported in the patients treated at the pulmonary department, and the 1-year survival rate in this group was over 91%. The authors concluded that pulmonary exacerbations in CF can be safely managed in a pulmonary department via NIV, provided the medical team is experienced on the CF and use of NIV (74).

In the early studies on the use of NIV in CF, it was stated that NIV is a short term bridge for patients waiting for lung transplantation. These studies showed that NIV avoids intubation related complications such as infections and barotrauma, facilitates weaning from mechanic ventilation in the post-operative period of transplanted patients, and reduces health care costs (75). Additionally, NIV has a place in the management of patients with end-stage cystic fibrosis lung disease, who are already on a transplant waiting list or who are being evaluated for lung transplantation (75). Although the effect of NIV on survival is controversial, it is a suitable treatment option in the palliative care of patients who are not eligible for transplantation (75). Non-invasive ventilation has also been increasingly investigated outside transplant-listed patients in the later studies (76, 77).

Although there is no data in children, studies in adults showed the benefits of NIV in preservation of lung functions in advanced CF patients. Fauroux et al. evaluated long-term NIV among adult CF patients with severe lung disease, 41 patients (male/female ratio = 20/21) with advanced lung disease on NIV were compared to 41 age and gender matched controls. The ventilated group was compared to the control group at year −1, during the year of NIV initiation (year 0) and 1 year after NIV (year +1) Mean age was 23 ± 8 years in both groups. The two groups had a similar level of mean FVC 43.7 ± 11.6 and 49.1 ± 15.4 and FEV_1_ at year −1. (28.2 ± 10.0 and 28.5 ± 10.1% for the ventilated group and control group, respectively). At year 0, the ventilated group had significantly greater declines in FVC (−3.6 ± 9.2 vs. +0.8 ± 8.9%, *p* = 0.03) and in FEV1 (−3.0 ± 6.7 vs. +2.6 ± 4.4, *p* < 0.0001). At year +1, the decreases in FVC (−2.1 ± 10.0 vs. −2.2 ± 9.9%) and FEV1 (−2.2 ± 6.7 vs. −2.3 ± 6.2%) were similar in both groups. Although the fall of lung function was greater in the ventilated group prior to the NIV use, NIV was associated with the stabilization of the decline in lung function in the ventilated group after 1 year of follow-up (76). In a more recent retrospective study, patients with advanced lung disease who received long-term NIV were investigated. 47 patients (30 males) were included to study. The mean age was 29 years, and the mean FEV_1_ was 20.7% predicted. The analysis of clinical data of patients at 12, 24, and 36 months before and after initiation of NIV revealed that 24 of 33 subjects showed an improvement in lung function or a drop in the rate in the deterioration of lung function. The mean duration of NIV use was 16 months in this study. Although the improvement in respiratory functions was most remarkable in the 1st year, this effect was observed to continue in patients who used NIV over 3 years. (FEV_1_ and FVC increased at the 1st year, 18 and 69 mL, respectively. At the 3rd year the increase in FEV1 and FVC was 43 and 142 mL, respectively). The authors emphasized that NIV may slow or reverse the decline in lung function in adults with advanced CF (77). However, the authors did not record the data of adherence to NIV in either of the studies (76, 77).

NIV can be also used for overnight ventilation in CF patients. There are many studies reporting sleep-disordered breathing (SDB) in patients with CF. A meta-analysis showed that CF patients had lower O_2_ saturation levels, poorer sleep efficiency than healthy controls, but higher respiratory event indices. This study also revealed a positive correlation between nocturnal desaturation and disease severity. Sleep is negatively affected by several factors in CF patients (78). Chronic lung disease-related gas-exchange abnormalities, chronic nocturnal cough, gastroesophageal reflux, abdominal pain may cause sleep disruption and SDB in CF patients. Additionally, upper airway disease and impaired mucociliary activity may lead to obstructive sleep apnea syndrome (11). Early diagnosis and treatment of SDB may prevent cardiopulmonary complications such as pulmonary hypertension, improve pulmonary outcome, as well as psychological and cognitive health (11, 78).

According to the CF Foundation recommendations, oxygen supplementation is recommended for patients with nocturnal hypoxemia, while NIV should be considered in cases with hypercarbia (68). Nocturnal implemented NIV can prevent the fall in minute ventilation from NREM to REM sleep in patients with CF (62).

Milros et al. investigated ventilation in all sleep stages in 13 CF patients (aged 26 ± 5.9 years) with moderate to severe lung disease and compared the results of low-flow oxygen and NIV on ventilation and gas exchange during sleep. They found that minute ventilation in REM sleep was significantly lower than in NREM sleep or wakefulness. Both low-flow oxygen and NIV increased the nocturnal oxygen saturation level. The measure of transcutaneous carbon dioxide levels revealed that the maximal change in transcutaneous carbon dioxide levels from NREM to REM with low-flow oxygen was 4.7 ± 2.9 vs. 1.6 ± 0.9 mmHg on NIV. Thus, they showed that the initiation of BIPAP decreases REM related hypoventilation (79). Patients, who were already receiving supplemental oxygen therapy, showed further improvements in nocturnal ventilation with the addition of NIV (80, 81). Another prospective, randomized, parallel-group study in adult patients with CF and sleep desaturation was recently conducted. This study compared 12 months of NIV (±O_2_) to long term oxygen therapy. There were 14 patients (mean age = 28 years) in the NIV (±O_2_) group and 15 patients (mean age = 31 years) in the long term oxygen group. Unlike other studies, NIV (±O_2_) during sleep was associated with event-free survival, which defined as no deterioration in awake CO_2_ levels, transplantation, or death, over 12 months in adults with CF (82). It should be noted that since these studies are conducted among adults, these results should be evaluated in the context of using criteria specifically established for children for example different criteria used to define hypoventilation during sleep.

A recent Cochrane review also stated that NIV, in combination with oxygen therapy improves gas exchange during sleep to a greater extent than oxygen therapy alone in people with moderate to severe CF lung disease in single night studies (83).

However, based on studies of polysomnographic data in CF patients, total sleep time, sleep efficiency, and arousals remained similar despite the use of NIV (11). But exercise capacity and scores of health-related quality of life also showed an improvement with the implementation of NIV in patients with CF (11).

NIV is also usefull as an adjunct to other airway clearance techniques, particularly in people with cystic fibrosis who have difficulty expectorating sputum (83). Studies evaluating the impact of NIV on airway clearance therapy showed that the use of NIV improved respiratory muscle performance, oxygen saturation levels and pulmonary lung functions measured by lung clearance index during a 3-month period (84, 85). Faouroux et al. investigated the effect of NIV on respiratory muscle fatigue and oxygen desaturation during chest physiotherpy in children with CF. Authors measured maximal inspiratory and expiratory pressures before and after chest physiotherapy in CF patients, who received positive pressure only during inspiration, and found a decrease in respiratory muscle performance and in oxygen saturation level. They demonstrated an improvement in muscle fatigue and desaturation with the implementation of NIV during chest physiotherapy. These results may be explained by the effect of NIV as an external inspiratory muscle, and increased lung and chest wall compliance. Non-invasive ventilation may be used with chest physiotherapy in patients with muscle fatigue and desaturation and poor tolerance of the chest physiotherapy sessions (84). Although there are no specific criteria for timing of addition of NIV to chest physiotherapy in children with CF, it may be considered in children with an acceleration of lung function decline requirement admissions. It has been shown that NIV enhances aerosol deposition in cystic fibrosis, but there are no studies investigating the integration of NIV and chest physiotherapy with nebulized treatments (25).

A study evaluating the practice on the use of NIV across pediatric CF centers in United Kingdom and Australia showed that only 0.4% percent of all patients were on NIV therapy. NIV was initiated according to a protocol only in 30% of centers. The most frequently used methods of NIV were BIPAP via nasal masks. Sleep studies were performed in less than half of the centers for the decision of initiation or follow up of NIV, and the pulse oximetry was the most used method for evaluation (86).

As discussed above, there is a physiological rationale for the use of NIV in patients with CF lung disease. However, long-term randomized controlled trials are required to determine the clinical effects of NIV and to establish validated criteria to start NIV in children with cystic fibrosis.

## Interstitial Lung Disease

Interstitial lung disease represents a heterogeneous group of rare respiratory disorders and is characterized by the presence of diffuse infiltrates on lung imaging, and abnormal pulmonary function tests indicating a restrictive ventilatory defect and/or impaired gas exchange due to inflammatory and fibrotic changes of the lung parenchyma (87). A recent classification based on the patient's age, clinic findings, and radiologic and histologic characteristics have been developed. Interstitial lung diseases are grouped as ILD specific to children aged <2 years and as ILD not specific to age. While surfactant protein deficiency, neuroendocrine cell hyperplasia of infancy, pulmonary interstitial glycogenesis, and developmental disorders are specific to infancy, ILD related to exposure/environment insults, ILD related to systemic and immune diseases, and ILD related to primary lung parenchyma dysfunctions constitute the non-age specific group (88). Anti-inflammatory and immunosuppressive agents are the cornerstones of pharmacological therapy, while oxygen and ventilatory support are required for patients with hypoxemia and respiratory failure (9).

In ILD, increased respiratory rate, increased resistance, decreased lung compliance increase work of breathing, and respiratory load. Non-invasive ventilation is expected to correct these pathological processes, but the results are controversial in ILD patients. In adult studies, the success rate of NIV in chronic restrictive diseases was lower compared to obstructive diseases. This difference was linked to a markedly reduced inspiratory flow and tidal volumes due to decreased compliance (89, 90).

There are no studies on the NIV use among children with ILD. The study investigating 1-year outcomes of the children with a new diagnosis of ILD, submitted to the ChILD-EU Registry revealed that over 12 months, the number of the children receiving NIV was close to children receiving invasive mechanical ventilation (32 vs. 34%) (91).

Current data on the NIV use in ILD is limited to adult studies. The most common indication of NIV is the acute respiratory failure (ARF) episode of ILD. The outcome of ARF is poor in ILD patients. Due to the potential side effects of invasive mechanical ventilation, NIV has expected to be the preferred method in stabilizing ARF, minimizing the intubation related the complications. Studies evaluating the outcomes in ILD patients during an ARF episode requiring ventilatory support found that despite palliative improvement with NIV, the prognosis was poor (17, 92) Authors stated that the NIV should be considered in less severe patients with an acute physiology and chronic health evaluation (APACHE) II score >20 because of mortality risk and high unsuccessful rate (92). A recent study evaluating risk factors for mortality in ILD patients during an ARF episode requiring ventilatory support found that the survival was better for patients receiving NIV support compared to invasive mechanical ventilation (93). The etiology of acute respiratory failure (ARF) episode is also important on the NIV response. Aliberti et al. found an improvement in oxygenation during NIV treatment in patients with ARF due to pneumonia, but not in other etiologies such as fibrosis and other triggers responsible for ARF, and they suggested to individualize NIV treatment (94).

The use of NIV in ILD in chronic hypercarbic respiratory failure was reported by Koschel et al. in a case series. They used NIV in 10 adult patients during sleep, and they showed a decrease in daytime arterial pressures of carbon dioxide and an increase in the arterial partial pressure of oxygen (95).

NIV can also be used during chest physiotherapy sessions in patients with ILD. Dreher et al. compared the 6-min walk distance (6MWD) and quality of life scores measured by short form 36 in hypercarbic ILD patients receiving nocturnal NIV patients to those who are normocarbic and not receiving NIV. They found an increase in the 6MWD and the mental score in the group who used NIV. Authors concluded that nocturnal NIV was able to improve exercise capacity and quality of life (96).

Finally, NIV may also be considered in palliative care in ILD patients. A retrospective analysis revealed that NIV was used in 29% of the ILD patients to relieve dyspnea in patients with end-stage lung diseases in 6 months prior to the death (97).

## Non-Cystic Fibrosis Bronchiectasis

Non-Cystic fibrosis bronchiectasis is one of the most common causes of chronic lung diseases. Typical findings are persistent or recurrent (>3) episodes of chronic wet or productive cough, coarse crackles and digital clubbing, and the presence of bronchial dilation in high-resolution chest tomography (HRCT) (98). There is an obstructive pattern in pulmonary function tests, a concomitant restrictive pattern may also occur. As the disease progresses, there is an increase in the ventilation/perfusion mismatch, and hypoxemia followed by the development of hypoventilation and hypercapnia. Infections, immune deficiencies, primary ciliary dyskinesia (PCD), and recurrent aspirations are the main causes of non-CF bronchiectasis. Between 19 and 55% of the patients with non-CF bronchiectasis may be considered idiopathic as underlying etiology may not be identified (98, 99). The objectives of the management in non-CF bronchiectasis are the treatment of respiratory infections and augmentation of airway clearance with chest physiotherapy to prevent the decline in lung functions and to improve quality of life (98, 99).

The data on the use of NIV in children with non-CF bronchiectasis is scarce. According to a retrospective adult study investigating trends in NIV use and outcomes in COPD and non-COPD patients with acute respiratory failure over 15 years showed that the number of patients with severe bronchiectasis requiring NIV is decreased. This study also revealed that the efficacy rate was lower in the non-COPD group (bronchiectasis, ILD) compared to COPD patients (100).

NIV is increasingly used in non-CF bronchiectasis patients with acute respiratory failure. The adult studies investigating the efficacy of NIV in non-CF bronchiectasis patients with acute respiratory failure due to exacerbations or other etiologies such as pneumonia showed comparable results between NIV and invasive mechanical ventilation. A retrospective study investigating the results of NIV and invasive mechanical ventilation use among 99 patients with ARF and non-CF bronchiectasis revealed that NIV was used in 67% of the patients, and the success rate was 65%. The efficacy in correcting blood gases was similar in both groups. Also, the total duration of stay in the hospital was similar in both groups. The authors concluded that the NIV use is a feasible treatment option for the management of ARF with non-CF bronchiectasis (101).

Long term use of NIV was investigated in adults with non-CF bronchiectasis and chronic respiratory failure. Benhamou et al. studied long-term efficacy and tolerance of NIV in 14 patients with non-CF bronchiectasis and chronic respiratory failure and compared with those who received long-term oxygen support only. NIV use was associated with the reduction of the days of the hospitalization, but partial oxygen saturation and the overall survival rates were similar in both groups. Non-invasive ventilation was well-tolerated in 11 of 14 patients (102).

In another retrospective study on the long-term effects of NIV in adults with non-CF bronchiectasis and chronic respiratory failure, blood gas levels, duration of hospitalization, and questionnaires to determine the patients' perception of the benefits of the treatment were evaluated before and after the initiation of NIV. The median duration of NIV use was 26 months. With the initiation of NIV, a stabilization in PCO_2_ levels was recorded. Duration of hospitalization between 12 and 24 months after starting NIV was decreased compared to 1 year before the initiation of NIV. An improvement in the quality of life was also observed (103).

## Bronchiolitis Obliterans

Bronchiolitis obliterans is a rare chronic lung disease characterized by obstruction of the small airways. Bronchiolitis obliterans is mainly caused by infections, inhalation of toxic fumes, connective tissue diseases, and after hematopoietic stem cell transplantation. The main histological findings of BO are fibrosis and inflammation of terminal and respiratory bronchioles (104, 105). Tachypnea, hypoxemia, and persistent wheezing are common symptoms. It has been also reported that children with BO also have sleep-disordered breathing, and increased risk of nocturnal hypoxemia which is correlated with the severity of lung functions (106). Prognosis and treatment differ in BO patients based on the etiology. While patients with post-infectious BO have chronic and slowly progressive course, other etiologies may cause a rapidly progressive disease.

There is still no evidence regarding treatment and the mechanisms of the development of BO in children. The current management of BO is based on the studies among adult BO patients mainly caused by lung transplant and hematopoietic stem cell transplantation. The management includes systemic and nebulized anti-inflammatory agents, nebulized bronchodilators in addition to general supportive care including respiratory support, pulmonary physiotherapy, and nutritional support, and children with BO should be followed up by a multidisciplinary team in specialized centers (105).

There is no study on the application of NIV in post-infectious BO patients. In a case report, the importance of the analysis of the work of breathing and respiratory mechanics to optimize of NIV treatment for BO was addressed. NIV following physiologically determined settings, reduced hyperinflation, decreased respiratory rate, and normalized the inspiratory time/total duty cycle ratio in an infant with postinfectious BO, which resulted in a decrease of work of breathing (107).

## Complications and Adherence

There are no specific references for compliance and adherence of NIV in children with chronic lung disease. Interface-related complications are common in the use of long term NIV. Skin trauma caused by prolonged use of masks is the most common complication. Skin lesions were reported in half of the patients after 6-month use of NIV via nasal masks (108). Eye-irritation caused by air leaks may also occur due to inappropriate sizes of masks. In small children, nasal or facial masks can cause mid-face hypoplasia and malocclusion (109). Additionally, it can lead to neurodevelopmental delay by acting as a physical barrier to social interaction. The development of newer interfaces such nasal pillows may cause fewer complications with minimum pressure points (4). Mask fitting and strap tension should be optimized to avoid skin trauma. Alternating interfaces and use of protective skin gel pads are also useful (62, 109). Nasal pillows, nasal masks, nasobuccal masks, total face masks, and mouthpieces are interfaces used in NIV for children. From those, nasal pillows, which provide minimal skin contact and enhanced comfort, can be used for children aged 5–7 years or older (4).

Other complications are associated with the delivery of positive pressure. Gastric distention, gastroesophageal reflux, and feeding intolerance can be seen with high pressures. Another associated risk is pulmonary aspiration caused by vomiting while wearing an oronasal or face mask. High pressures may also lead to nasal congestion. These complications can be managed by decreasing the airway pressures and using nasal masks (4, 62).

Adherence to NIV is one of the major barriers to treatment in children. Currently there is no specific definition for adherence in children. Adherence definitions used in child studies in the literature are generally adapted from adults, although the sleep structure and duration of children are different from adults. There are several factors affecting adherence, including patient characteristics, devices and masks used, pressure settings, side effects, and psychological and social factors (110). Educational and behavioral interventions may improve adherence, peer support groups and individualized strategies are also recommended (109, 110).

Ramirez et al. investigated adherence to NIV in 62 patients (mean age, 10 ± 5 years) with three underlying disease categories including five children with chronic lung disease. Mean objective adherence was extremely high in that study with a mean use of 8.17 ± 2.30 h per night and 72% of the patients using their CPAP or NIV >8 h per night. Treatment adherence was not correlated to the type of underlying disease or the efficacy of NIV assessed on the nocturnal gas exchange. Authors commented that the excellent level of compliance in their population may be explained by the fact that NIV was initiated and followed in a dedicated pediatric NIV unit (111).

Another study evaluating NIV adherence, adherence barriers in patients with DMD using nocturnal NIV found that mask discomfort was the most commonly reported adherence barrier. The assessment of the psychosocial functioning of patients and their caregivers revealed that caregivers' barriers and child internalizing symptoms predicted lower NIV adherence, and authors recommended multidisciplinary approach to improve adherence in children (112).

## Conclusion

NIV is an effective treatment for chronic respiratory failure and sleep-disordered breathing including hypoventilation in children with chronic lung diseases. NIV reduces the need for invasive ventilation in some incidences and allows children to live with their families at home. The use of NIV in children with chronic lung disease is increasing worldwide, as a result of improvements in the survival rates of these children. NIV can be effective for acute asthma exacerbations in children. Children with BPD also could benefit from NIV support however no differences on long term benefits have been found between CPAP and NIV. There are adult studies supporting the use of NIV in patients with bronchiectasis and ILD. However, there are no studies investigating the effect of long-term NIV use among children with chronic respiratory diseases, except CF, which shows that the application of NIV results in the stabilization of the decrease in lung functions in advanced lung disease (76). Also, NIV may be a useful adjunct to physiotherapy in CF children. The heterogeneity of the diseases and ages of the patients requires close monitoring by an experienced team. Complications are mainly related to interfaces and positive pressure and these side effects can often be managed by applying the appropriate interfaces and settings. This approach is also important to improve the adherence. Limited number of contraindications such as severe cystic/cavitatic lung disease should also be kept in mind during evaluating the suitability of the patients for NIV. Future studies are required to determine the main criteria for NIV use and long- term benefits for these patients including the effect on the quality of life.

## Author Contributions

EA wrote the manuscript with support from RE. UK and RE reviewed and supervised the manuscript. All authors contributed to the manuscript and approved the submitted version.

## Conflict of Interest

The authors declare that the research was conducted in the absence of any commercial or financial relationships that could be construed as a potential conflict of interest.
